# Dissociable Processes for Orientation Discrimination Learning and Contextual Illusion Magnitude

**DOI:** 10.1371/journal.pone.0103121

**Published:** 2014-07-25

**Authors:** Charlotte Elizabeth Holmes Wilks, Geraint Rees, Dietrich Samuel Schwarzkopf

**Affiliations:** 1 Experimental Psychology, University College London, London, United Kingdom; 2 UCL Institute of Cognitive Neuroscience, London, United Kingdom; 3 Wellcome Trust Centre for Neuroimaging, University College London, London, United Kingdom; University of Muenster, Germany

## Abstract

Previous research suggests an inverse relationship between human orientation discrimination sensitivity and tilt illusion magnitude. To test whether these perceptual functions are inherently linked, we measured both orientation discrimination sensitivity and the magnitude of the tilt illusion before and after participants had been trained for three days on an orientation discrimination task. Discrimination sensitivity improved with training and this improvement remained one month after the initial learning. However, tilt illusion magnitude remained unchanged before and after orientation training, at either trained or untrained orientations. Our results suggest that orientation discrimination sensitivity and illusion magnitude are not inherently linked. They also provide further evidence that, at least for the training periods we employed, perceptual learning of orientation discrimination may involve high-level processes.

## Introduction

Visual illusions dissociate a physical stimulus from its subjectively perceived quality. This makes them popular tools for studying the neural processes associated with the contents of consciousness. For example, the tilt illusion is a contextual modulation of visual orientation [1], [2] that occurs when an oriented test grating is surrounded by an inducing grating. When this inducer is tilted approximately 15° relative to the test it causes the central grating to be perceived as tilted in the opposite direction [3].

We recently used the tilt illusion to study how the surface area of early visual cortices (V1, V2 and V3) is related to perception [4] because this illusion has been linked to cortical distance in visual cortex [5]. In that earlier study, we measured the magnitude of the tilt illusion and orientation discrimination sensitivity and found that both correlate with the retinotopically-defined surface area of V1. Specifically, individuals who are better at discriminating orientation exhibit weaker illusion magnitudes and also have greater V1 surface areas. This is consistent with the hypothesis that V1 surface area influences perception through the scaling of intra-cortical connections. That is, when the surface area of V1 is larger, intra-cortical connections may be weaker because there are physical constraints on their length and/or the speed of transmission. This would reduce the effect of contextual interactions and hence reduce illusion strength. Enhanced orientation discrimination in individuals with larger V1 could further result from differences in microscopic circuitry. Neurons in regions with more homogeneous orientation preferences show more selective tuning [6]. Since orientation is encoded in an orderly map comprising homogeneous orientation domains [7], [8], [9], it is possible that such domains are wider, and thus locally more homogenous, in individuals with larger V1. This would predict finer orientation selectivity on average and could result in greater discrimination ability.

However, another possibility is that poorer orientation discrimination trivially leads to larger illusion magnitudes. An observer bad at distinguishing very small physical differences in orientation may also tend to judge illusory orientation differences as larger because they overestimate the *perceived* difference in contextual effects like the tilt illusion. Rather than simply being poor at discriminating illusory differences, most participants presumably experience the tilt illusion and, while the measured illusion strength varies widely between observers, they thus agree that there is a perceived difference in orientation. In turn this could lead those individuals with worse discrimination ability to exaggerate the illusion.

Orientation discrimination is susceptible to perceptual learning, that is, training the discrimination task can have marked improvement in sensitivity that can be highly stimulus specific [10], [11], [12], [13]. If tilt illusion magnitude and orientation discrimination sensitivity were inherently and trivially linked, then improving discrimination would result in a concomitant reduction in illusion strength. In the light of our previous findings linking both discrimination ability and illusion strength to V1 surface area we sought to now test whether the relationship between illusion strength and discrimination sensitivity is preserved when discrimination improves. We measured both of these factors in normal healthy human observers, before and after three days of training on an orientation discrimination task.

## Materials and Methods

### Participants

Participants gave written informed consent to participate in the study. All procedures were in accordance with the Declaration of Helsinki and were approved by the University College London (UCL) Research Ethics Committee.

Eight participants took part in the first experiment (6 female, aged 20–28), and nine in the second (2 female, age 18–29). These participants were recruited from the UCL Psychology Subject Pool and gave written informed consent to participate in the study. All were right handed with no neurological problems and had normal or corrected-to-normal vision. An additional three participants had been recruited but were excluded from all analyses. One participant in experiment 1 stopped complying with task instructions during training. Two participants in experiment 2 misunderstood the task instructions in the pre-test and therefore did not provide any useful data. Since we aimed for participants to have a controlled exposure to our stimuli and task it was impossible to retest these participants.

### Stimuli

**Figure 1:**
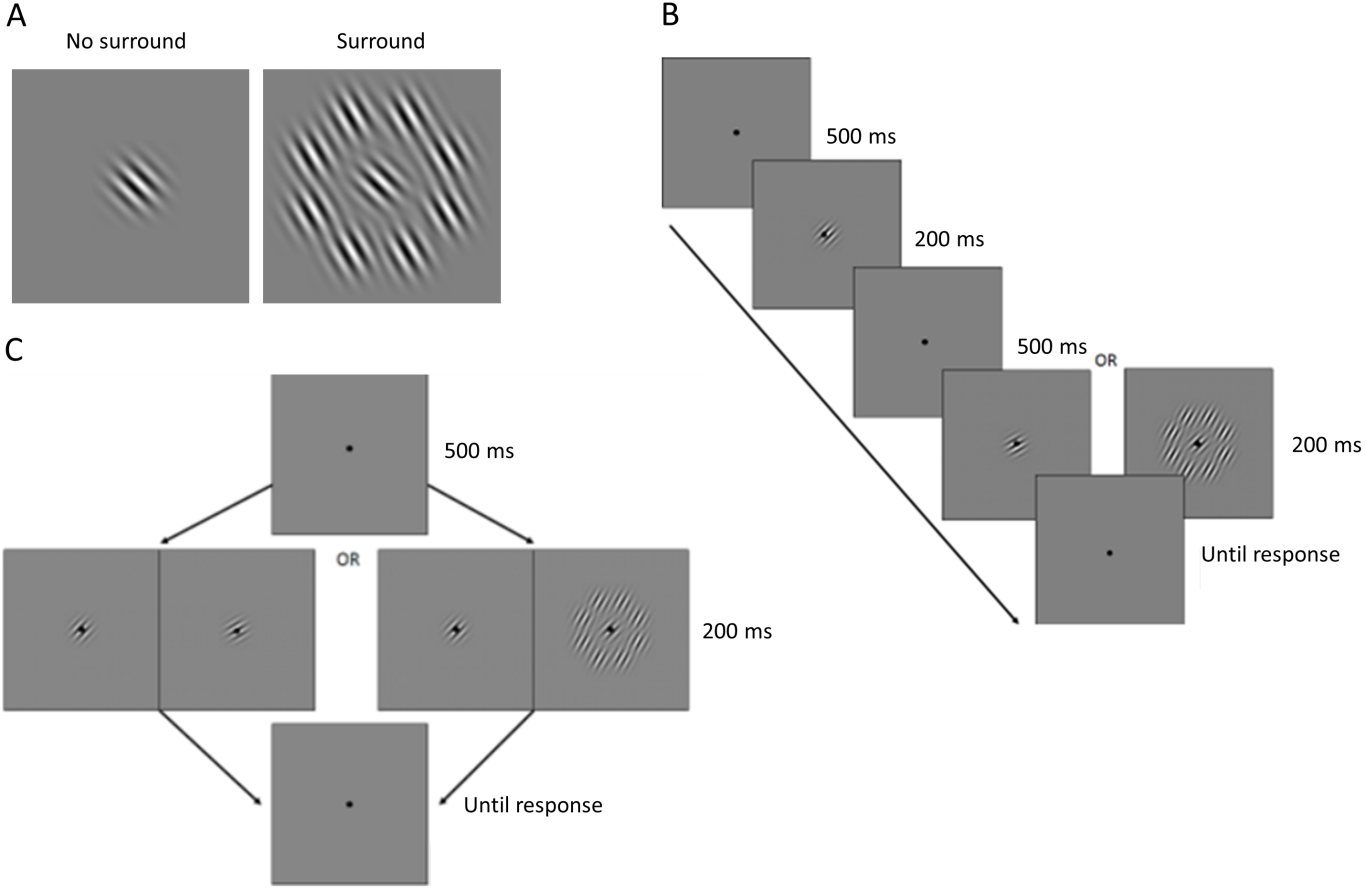
Tilt illusion and experimental design. A. Example of tilt illusion stimuli used in this study. The orientation of the central grating surrounded by an annulus of gratings is identical to the orientation of the grating on the right. However, due to the contextual interaction between center and surround it is perceived as tilted away from the surround orientation. B. Trial sequence in experiment 1. After a brief fixation period a reference grating appeared (either 45° or 135°), followed by another fixation period and the test grating, which could be surrounded by an annulus of grating patches (illusion stimulus) or be presented on its own. After this interval, participants were required to respond whether the test grating was tilted clockwise or anticlockwise relative to the reference. C. Trial sequence in experiment 2. After a brief fixation period, two stimuli appeared on either side of fixation. One was the reference grating (either 45° or 135°) while the other was the test grating, which could be surrounded by an annulus of grating patches (illusion stimulus) or be presented on its own. After this interval, participants were required to respond whether the left or the right grating stimulus appeared more vertical.

Participants were presented with Gabor patches, sinusoidal gratings convolved with a Gaussian envelope (standard deviation: 0.62° of visual angle, carrier wavelength: 0.41°) at maximal contrast against a uniform grey background. To measure orientation discrimination performance single Gabor patches were presented. To measure the magnitude of the tilt illusion, the test grating would include a surrounding annular context. This context comprised eight Gabor patches, on an imaginary circle (radius: 2.07°) around the target grating, that were tilted 15° from the central test grating towards vertical, creating the tilt illusion (Figure 1A).

Stimuli were presented on a Samsung SM2233RZ LCD display at a distance of 78 cm. The maximum and minimum luminance levels were 230 and 0.6 cd/m^2^ respectively, and the contrast was set at maximum. The display was gamma-corrected. To stabilize head position, participants used a chin and forehead rest. Participants were told to fixate on a dot (0.12° diameter) positioned in the center of the screen, and to maintain fixation throughout the experiments.

### Procedure

Participants were tested in a darkened room. Both experiments comprised a test day (day 1), in which (after a short demo exercise) orientation discrimination sensitivity and the tilt illusion magnitude were measured. This was followed by three orientation discrimination training days in which each participant was trained on a specific orientation of either 45° or 135° (days 2–4). We chose the oblique orientations of 45° and 135° because stimuli at these orientations are harder to discriminate than those oriented vertically or horizontally [14], [15] allowing stronger illusions [16] and greater room for improvement in discrimination ability [17]. After the training sessions participants then underwent a second test day (day 5). The 5 sessions were performed on consecutive days whenever possible, and in the instances where there was a gap of a day or more between the last training session and the last test session, participants performed 3–5 of the training blocks to re-accustom themselves with the task. In experiment 1 only, participants underwent a third test session approximately one month after the initial test session.

### Experiment 1

A test session for experiment 1 consisted of 20 blocks, each containing 44 trials, and took participants 35–60 minutes. During these sessions the orientation discrimination sensitivity and the magnitude of the tilt illusion were measured using the method of constant stimuli. After a short blank period during which the fixation dot was presented for 500 ms, a reference grating with a constant orientation of either 135° or 45° anticlockwise from horizontal was presented for 200 ms. After another inter-stimulus blank interval of 500 ms a test grating rotated by up to +/−30° from the reference appeared for 200 ms. The participant judged whether the test stimulus was rotated clockwise or anticlockwise in comparison to the constant reference. There were 11 possible orientation differences between the reference and the test gratings, varying between −30° (towards horizontal) and +30° (towards vertical): −30°, −15°, −7.5°, −3.75°, −1.5°, 0°, 1.5°, 3.75°, 7.5°, 15°, or 30° relative to the reference.

The 500 ms interval between the two stimulus presentations (during which only a blank screen with a fixation point was shown) should have been too long for producing an apparent motion percept. While there may be a small possibility that participants perceived a motion signal induced by the second stimulus relative to their memory of the first (reference) stimulus (or that they used the ‘rotation’ as a strategy for solving the task) no participant reported that they used such a strategy. Moreover, since the method for measuring the illusion and discrimination performance were the same, this should have no bearing on our conclusions.

During test sessions we measured tilt illusion magnitude in exactly half of trials so the test grating would include the surrounding context. Participants were instructed to respond with the index and middle fingers of their dominant hand by pressing one of two buttons on a computer keyboard. For 200 ms the fixation point would then turn green for a correct response and red for an incorrect response, and the next trial would continue following this feedback. In trials where there was no correct response (0 degree rotation) the feedback was pseudo-randomized with a probability of 0.5. Figure 1B shows an example trial sequence for both conditions. All trial conditions were shown in a randomly interleaved order with equal probability.

A training session consisted of 20 blocks, each containing 40 trials, and took participants 25–50 minutes. Before the first training session each of the participants were randomly assigned to be trained at discriminating orientations around references of either 45° or 135°. All three training sessions for a particular participant were then performed for the same assigned reference orientation. The task was exactly as during the test sessions, except that participants were never presented with an illusion stimulus (i.e. with a surround) and the test stimulus never had the same orientation (0 degree rotation) as the reference.

### Experiment 2

Although unlikely, it would have been possible in experiment 1 that participants were remembering the reference orientation shown initially (which was the same throughout the training trials –135° or 45°) and therefore not attending adequately to the reference orientation across trials. Moreover, many participants found the task relatively difficult resulting in relatively large variability in estimates. To counteract that we provided feedback even in test sessions but it was unclear whether such feedback interfered with measurement of the point of subjective equality (PSE). Experiment 2 was therefore an improved design in which the reference and test stimulus were presented side by side to allow direct comparison and feedback was only given during the training sessions.

A test session for experiment 2 consisted of 25 blocks, each containing 44 trials, and took participants 40–60 minutes to complete. There was only one stimulus interval. After a 500 ms blank period, in which only the fixation dot was presented, a reference grating with a constant feature value of either 135° or 45° was presented for 200 ms on either the left or right of a central fixation point at an eccentricity of 4.15°. A test grating appeared simultaneously and would be rotated up to +/−30° relative to the reference. This test grating was presented on the opposite side of the fixation point to the reference grating. The side where the test was presented varied randomly throughout the session. The participant would make a two-alternative forced choice judging which of the two gratings appeared more vertical. As in experiment 1, when measuring the magnitude of the tilt illusion the test grating would include a surrounding annular context. An example trial sequence for experiment 2 is shown in Figure 1C.

A training session for experiment 2 consisted of 40 blocks, each containing 40 trials, and took participants 40–60 minutes. Before the first training session each of the participants were randomly assigned to be trained at discriminating orientations around 45° or 135°. All three training sessions for a particular participant were then performed for the same assigned orientation. During training sessions, only the test gratings without a surround were presented. Feedback during training sessions was given by changing the color of the fixation point.

### Psychometric curves

For illustration only, we generated group-level psychometric curves in each test and training session by plotting the choice probability, that is the proportion of trials (averaged across participants) that the test grating was perceived to be tilted more vertically than the reference grating, against the true orientation difference between the test and reference gratings. Negative and positive orientation differences represented instances in which the test grating was physically more horizontal or vertical than the reference grating, respectively. We fitted a cumulative Gaussian function to these data in MATLAB (MathWorks, Inc.) by minimizing the least squares of the residuals between fit and observed data.

The function had three free parameters: the amplitude determining the upper and lower level where the function asymptotes, the bandwidth determining the slope of the function, and the threshold determining the shift to the left or right. The magnitude of the tilt illusion is described by the threshold, that is, the PSE where the orientation of the reference and test gratings was perceived to be the same. The amplitude and bandwidth together represent performance on the orientation discrimination task. The amplitude reflects coarser differences in discrimination ability as it is determined by large orientation differences; it also includes attentional lapses in the presence of strong signals. The bandwidth reflects the ability to make fine orientation discrimination near the threshold.

For statistical comparisons, we further fit cumulative Gaussian psychometric curves to the data in each condition and session separately for each participant. This was done using the maximum likelihood procedure described by [18] implemented in the MATLAB toolbox PsigniFit. This procedure incorporates a lapse rate parameter accounting for incorrect trials, due to attentional lapses, in easy stimulus conditions. The lapse rate therefore accounts for effects that are neither related to the slope nor the PSE of the psychometric function. We then extrapolated the PSE from the curves for each participant and also estimated the slope of the function at this threshold point. The slope is a measure of discrimination sensitivity with steeper slopes indicating better discrimination. We then performed statistical comparisons using repeated-measures ANOVA and planned comparisons using paired t-tests at the group level to test whether illusion magnitude/PSE and discrimination sensitivity had changed specifically due to training.

We also estimated the minimal ‘theoretically relevant change’ in illusion magnitudes. Using our previous work demonstrating a correlation between discrimination thresholds and tilt illusion magnitudes [4], we estimated that the ratio between illusion magnitude and threshold performance at ∼71% correct is approximately 2∶1. Thus, if the relationship were maintained despite training we could expect the illusion to be reduced by twice the change in thresholds. In our present experiments the learning effect amounted to a reduction in thresholds of 3.9 degrees in experiment 1 and 2.3 degrees in experiment 2. Thus, if a similar relationship were maintained after training we would surmise that the illusion should be reduced by approximately 5–8 degrees.

## Results

### Experiment 1

**Figure 2:**
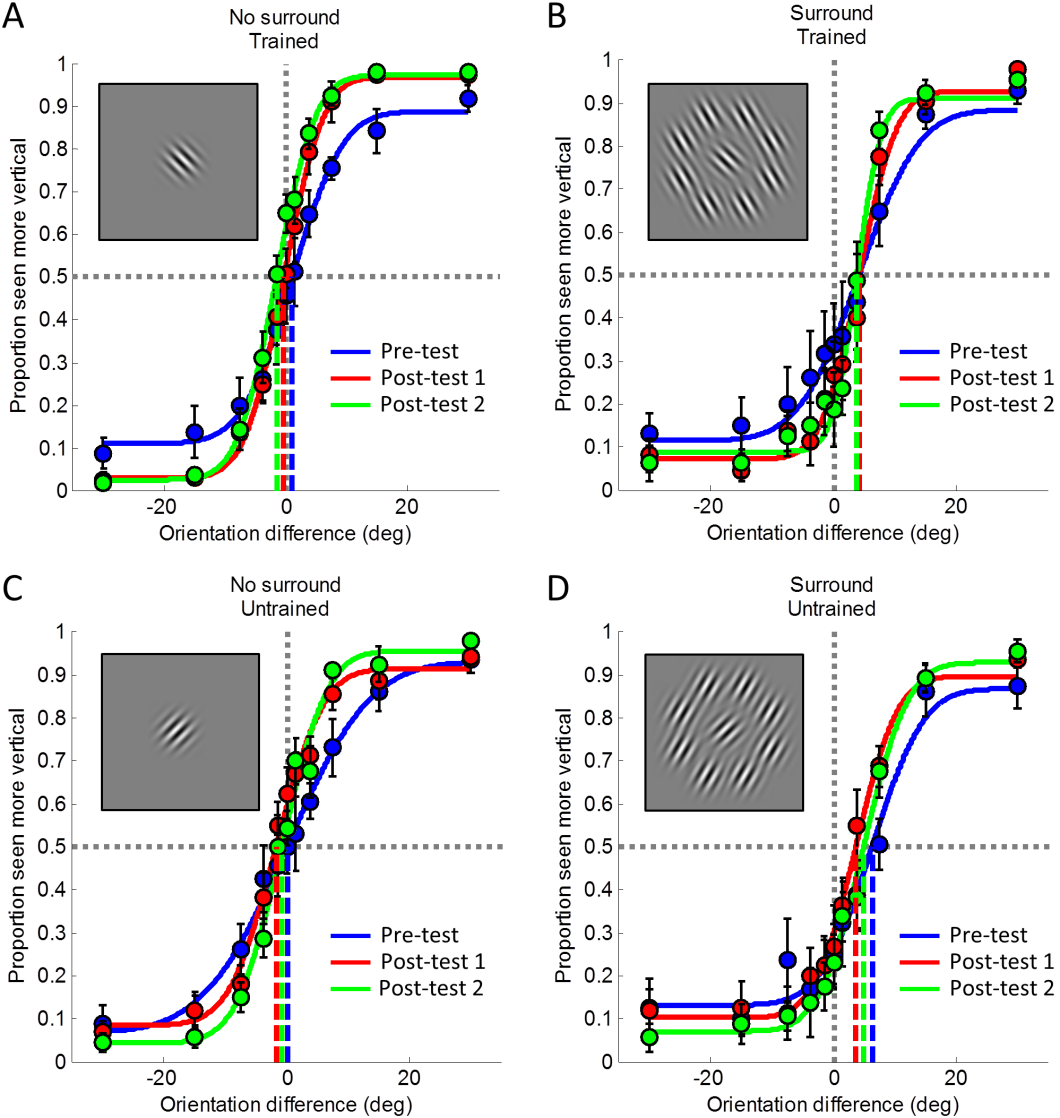
Psychometric curves from experiment 1. The proportion of trials that the test grating was seen as more vertical is plotted against the physical orientation difference between the test and the reference gratings. Each point denotes the average over participants; error bars indicate ±1 standard error of the mean. Solid curves show the cumulative Gaussian functions fitted to these data. Blue: pre-test, before training. Red: post-test 1, after training. Green: post-test 2, approx. one month later. A. Trained orientation, no surround. B. Trained orientation with surround (illusion stimulus). C. Untrained orientation, no surround. D. Untrained orientation with surround (illusion stimulus). The insets illustrate the stimulus conditions but note that which orientation was trained was counterbalanced across participants.

We measured both orientation discrimination sensitivity and the magnitude of the tilt illusion before and after training discrimination sensitivity. Figure 2 shows average psychometric curves in experiment 1 before (blue curves) and after training (red curves). The curves for all four conditions, but particularly for the trained orientation without a surround (Figure 2A), all became steeper after training, reflecting an increase in discrimination ability. Similarly, the asymptote levels also increased markedly. In contrast, the tilt illusion magnitude reflected by the right-ward shift of the psychometric curves for stimuli with a surround (Figure 2B, D) did not change substantially due to training, especially not for the trained orientation (Figure 2B). Psychometric curves also remained very stable even one month after the initial test session (green curves).

**Figure 3:**
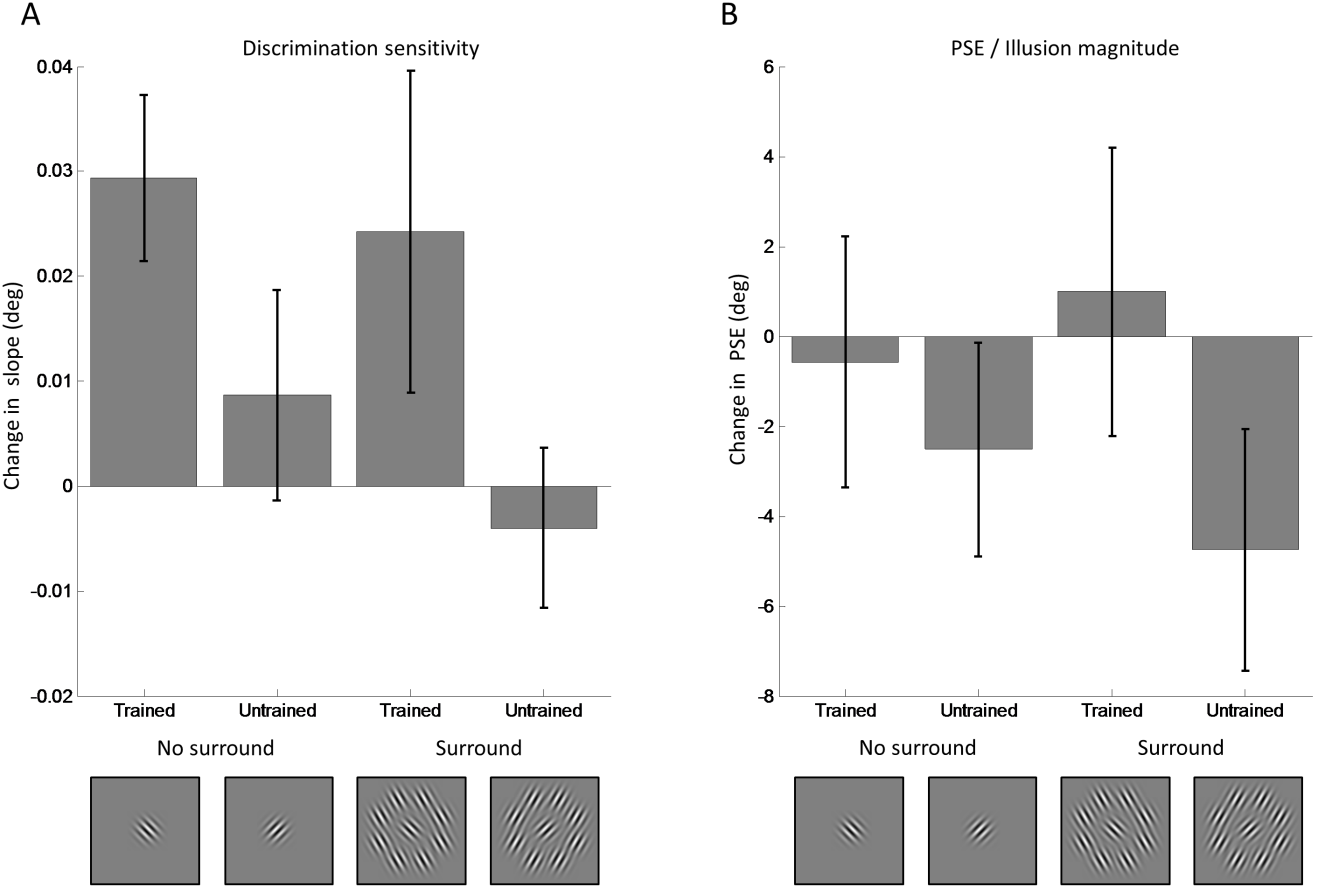
Learning effects in experiment 1. A. The effect training had on orientation discrimination in experiment 1 was quantified as the change in orientation discrimination sensitivity between test sessions 1 and 5 (the training effect). This was calculated by subtracting the slopes for the pre-test (session 1) from the slopes for the post-test (session 5) for each participant and then calculating the average across all participants. The training effect was significantly greater around the trained than the untrained reference orientations. B. Tilt illusion magnitude (with surround) and perceptual biases (without surround) were estimated by extrapolating the PSE from the psychometric curve fitted to the test sessions. We calculated the difference in PSE between session 1 and session 5 by subtracting the PSE for the pre-test (session 1) from the PSE for the post-test (session 5) and averaging these differences across all participants. Critically, illusion magnitude (which was bias corrected by subtracting the PSE without surround for each participant) did not change significantly between session 1 and session 5 at the trained or untrained conditions. In both panels error bars indicate ±1 standard error of the mean across participants. The insets illustrate the stimulus conditions but note that which orientation was trained was counterbalanced across participants.

The difference between the curves for the pre-test and first post-test revealed that discrimination sensitivity improved significantly for all stimulus conditions. This was supported by a 3-way repeated measures ANOVA (Table 1) over the slopes at the single participant level (Figure 3A) with the factors session (before and after training), training (trained or untrained reference orientation), and surround (with or without the surrounding annular context providing the tilt illusion). There was a significant interaction between training and session (Table 1). No other main effect or interaction reached significance.

**Table 1:** Three-way ANOVA on discrimination slopes in experiment 1.

Factor/Interaction	Degrees of freedom	F	p
Surround	1, 7	0.02	0.888
Trained	1, 7	5.31	0.055
Session	1, 7	4.47	0.072
Surround×Trained	1, 7	0.10	0.759
Surround×Session	1, 7	0.90	0.375
Trained×Session	1, 7	6.19	0.042*
Surround×Trained×Session	1, 7	0.18	0.685

*p<0.05.

It is important to test whether learning effects occurred for discrimination slopes on the trained conditions. Specifically, planned comparisons showed that discrimination sensitivity improved for the trained stimulus without surround (t(7) = −3.725, p = 0.007). With a surround this difference did not reach statistical significance (t(7) = −1.583, p = 0.157) although it showed an effect in the same direction. On the other hand, there was no significant training effect for the untrained orientation in either stimulus condition (no surround: t(7) = −0.869, p = 0.414, with surround: t(7) = 0.525, p = 0.616). Importantly, there was also a significant difference between the training effect for the trained and untrained orientation when no surround was present (t(7) = 2.402, p = 0.047). There were no other significant differences (all p’s>0.05). Taken together, these results suggest that perceptual learning occurred between the pre-test (session 1) and the post-test (session 5), because orientation discrimination sensitivity improved specifically at the trained orientation.

Importantly, the illusion magnitudes and PSE (Figure 3B), revealed by the PSE of the single participant curves, respectively, did not change significantly for any condition. This suggested that while performance on the orientation discrimination task improved, the illusion magnitudes remained stable. Critically, a 3-way repeated measures ANOVA revealed no change in illusion magnitude after training (Figure 3B). There were no significant main effects or interactions due to training for either the trained or untrained stimulus with a surround (Table 2). It did however show a strong main effect due to the presence of the surround confirming that a significant tilt illusion occurred with these stimuli but did not change with training.

**Table 2:** Three-way ANOVA on PSE in experiment 1.

Factor/Interaction	Degrees of freedom	F	p
Surround	1, 7	13.78	0.008*
Trained	1, 7	0.40	0.549
Session	1, 7	0.82	0.396
Surround×Trained	1, 7	3.44	0.106
Surround×Session	1, 7	0.05	0.836
Trained×Session	1, 7	1.27	0.297
Surround×Trained×Session	1, 7	1.38	0.279

*p<0.05.

In these analyses the PSE, or illusion magnitude (i.e. when the surround was present), was not corrected for perceptual bias when the surround, and hence the illusion, was absent. We found that the PSE without surround in the first test session was quite variable (untrained orientation: −7.80 to 11.98, trained orientation: −15.77 to 5.44), and represented individual perceptual bias independent of the illusion. This could have affected the results and therefore bias correction was performed by subtracting the PSE when no surround was present from the illusion strength (i.e. the PSE with surround) in both the pre and post-test data. The pattern of results, that is no change in illusion strengths due to training, was highly consistent with those without bias correction. In Figure 3B we show bias corrected data.

Our psychometric curve fitting also included a lapse rate parameter to account for a small level of incorrect responses when stimulus conditions made the task easy. We tested differences in lapse rates using the same procedure as for illusion strengths and discrimination slopes. It would not be surprising if lapse rates decreased with training as participants became more familiar with the task. However, we found no significant main effect or interaction for any factor with regard to lapse rates (all p’s>0.12).

In light of our previous results [4], we also tested whether there was a correlation between illusion strength and discrimination slopes in our pre-training data. While this relationship showed the expected direction it did not reach statistical significance (r = −0.24, p = 0.365). This is however unsurprising, because our within-subject design was underpowered for testing individual differences.

One month after the initial testing sessions we performed a second test session to examine whether the improvement in discrimination ability (observed after training) remained stable over this period. We therefore compared the orientation discrimination sensitivity in the original post-test session 5 (post-test 1) with that in an identical test session one month later (post-test 2). We did not find a significant difference between the orientation discrimination sensitivity in post-test 1 and that in post-test 2 for the trained orientation without a surround (t(7) = −0.568, p = 0.588) or across any of the other conditions (all p’s>0.1). This suggests that the perceptual learning for orientation discrimination was not a temporary phenomenon. For completeness, we also confirmed that the illusion strength (threshold in surround condition at trained orientation) also remained stable between post-tests 1 and 2 (t(7) = 0.32, p = 0.76) even though there had been no significant effect of learning for that initially.

Although the results of this experiment were conclusive, we decided to re-design the procedure in order to reduce the relatively large variability of PSEs without a surround, which may have contributed to the generally large improvement across all stimulus conditions after training. The task proved difficult for many of our participants as most were unfamiliar with psychophysics experiments and with the stimuli. One problem with experiment 1 may have been the temporal design that required participants to hold the reference orientation in memory before the test stimulus appeared. Alternatively, it is possible that participants did not fully attend to the constant reference in the first interval and merely compared stimuli to an internal reference, in particular after they had completed a number of trials and had thus been exposed to the reference repeatedly.

### Experiment 2

We therefore sought to improve participants’ general performance on the task before training and to require them to attend equally to the reference and the test stimuli by making a direct comparison between them. In experiment 2 we once again measured both orientation discrimination sensitivity and the magnitude of the tilt illusion before and after training to improve discrimination sensitivity. Participants underwent two test sessions (days 1 and 5) and 3 training sessions (days 2–4) as in experiment 1. However, instead of a temporal interval design, participants were now presented with a reference and a test stimulus simultaneously, at spatially separate locations (left and right of fixation), and were instructed to judge which of the stimuli appeared to be more vertical.

**Figure 4:**
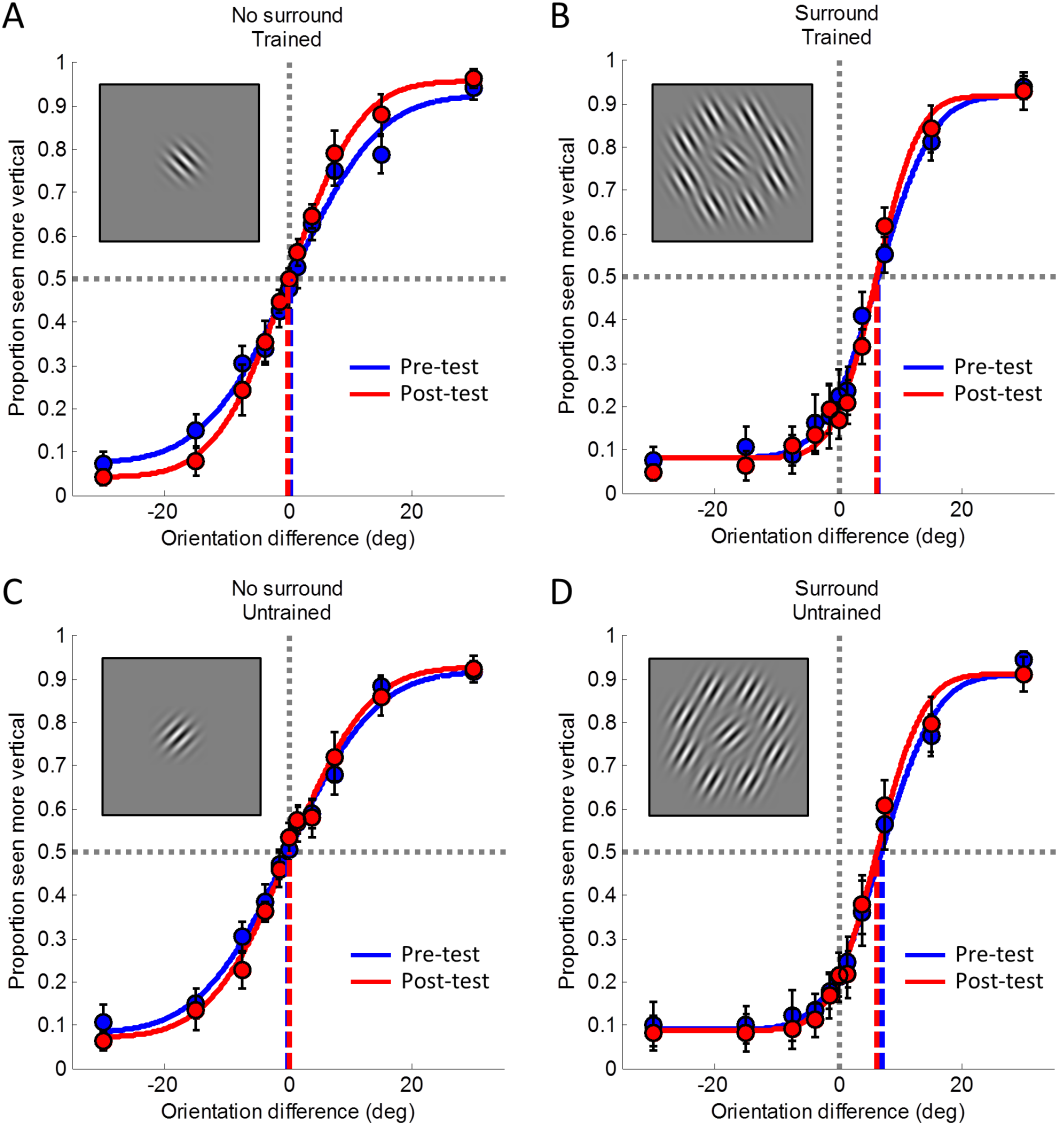
Psychometric curves from experiment 2. The proportion of trials that the test grating was seen as more vertical is plotted against the physical orientation difference between the test and the reference gratings. Each point denotes the average over participants; error bars indicate ±1 standard error of the mean. Solid curves show the cumulative Gaussian functions fitted to these data. Blue: pre-test, before training. Red: post-test 1, after training. A. Trained orientation, no surround. B. Trained orientation with surround (illusion stimulus). C. Untrained orientation, no surround. D. Untrained orientation with surround (illusion stimulus). The insets illustrate the stimulus conditions but note that which orientation was trained was counterbalanced across participants.

Figure 4 shows group average psychometric curves for the stimulus conditions in experiment 2. As in experiment 1, the curve for the trained orientation without a surround became steeper after training (Figure 4A). There was also a subtle increase in the asymptote level. However, for the other stimulus conditions (Figure 4B–D) there were no marked differences before and after training.

**Figure 5:**
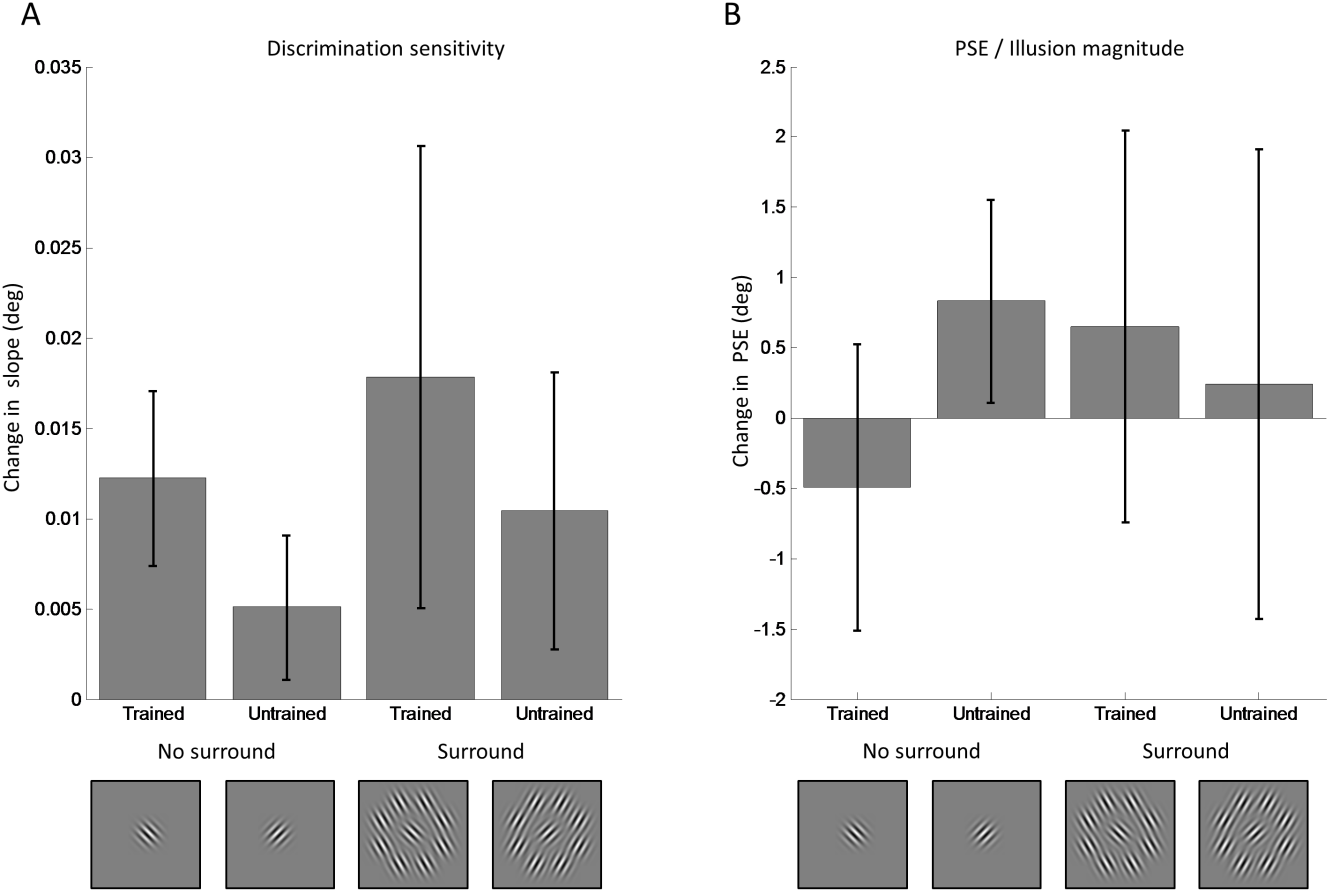
Learning effects in experiment 2. A. The effect training had on orientation discrimination in experiment 2 was quantified as the change in orientation discrimination sensitivity between test sessions 1 and 5 (the training effect). This was calculated by subtracting the slopes for the pre-test (session 1) from the slopes for the post-test (session 5) for each participant and then calculating the average across all participants. The training effect was significantly greater around the trained than the untrained reference orientations. B. Tilt illusion magnitude (with surround) and perceptual biases (without surround) were estimated by extrapolating the PSE from the psychometric curve fitted to the test sessions. We calculated the difference in PSE between session 1 and session 5 by subtracting the PSE for the pre-test (session 1) from the PSE for the post-test (session 5) and averaging these differences across all participants. Critically, illusion magnitude (which was bias corrected by subtracting the PSE without surround for each participant) did not change significantly between session 1 and session 5 at the trained or untrained conditions. In both panels error bars indicate ±1 standard error of the mean across participants. The insets illustrate the stimulus conditions but note that which orientation was trained was counterbalanced across participants.

As in experiment 1 a 3-way repeated measures ANOVA confirmed that orientation discrimination sensitivity (Figure 5A) was significantly greater after training. There was a significant main effect of session and also a significant main effect of surround (Table 3). Planned comparisons using paired t-tests confirmed a significant difference between the slopes at the trained orientation before and after training when no surround was present (t(8) = −2.53, p = 0.035). A similar change was observed when a surround was present, although again this effect did not reach statistical significance (t(8) = −1.39, p = 0.201). There was no significant training effect for the untrained orientation (no surround: t(8) = −1.27, p = 0.239, with surround: t(8) = −1.36, p = 0.210). Importantly, there was almost a significant difference between the training effect for the trained and untrained orientation when no surround was present (t(8) = 1.92, p = 0.091). Taken together, these results suggest that (consistent with our earlier findings in experiment 1) perceptual learning occurred between the pre-test (session 1) and the post-test (session 5), because orientation discrimination sensitivity was greater after training.

**Table 3:** Three-way ANOVA on discrimination slopes in experiment 2.

Factor/Interaction	Degrees of freedom	F	p
Surround	1, 8	8.09	0.022*
Trained	1, 8	1.52	0.253
Session	1, 8	8.62	0.019*
Surround×Trained	1, 8	0.01	0.926
Surround×Session	1, 8	0.75	0.412
Trained×Session	1, 8	0.81	0.395
Surround×Trained×Session	1, 8	0.00	0.990

*p<0.05.

Again and critically, a 3-way repeated measures ANOVA revealed that there was also no change in illusion magnitude after training (Figure 5B) for either the trained or untrained stimulus with a surround (Table 4). However this did show a strong effect due to the presence of the surround, confirming that a significant tilt illusion occurred but it did not change with training.

**Table 4:** Three-way ANOVA on PSE in experiment 2.

Factor/Interaction	Degrees of freedom	F	P
Surround	1, 8	29.43	0.001*
Trained	1, 8	0.05	0.828
Session	1, 8	0.19	0.677
Surround×Trained	1, 8	0.28	0.613
Surround×Session	1, 8	0.02	0.880
Trained×Session	1, 8	0.55	0.480
Surround×Trained×Session	1, 8	0.97	0.353

*p<0.05.

Illusion magnitude/PSE data were not bias corrected. The spatial two-alternative forced choice design was successful at minimizing bias in the no surround condition. However, for completeness we bias corrected the data by subtracting the PSE without surround from the illusion strengths (PSE with surround) for each participant. As in experiment 1, bias correction did not qualitatively alter the overall pattern of our results. The data shown in Figure 5B have been bias corrected.

As in experiment 1, we found no significant correlation between illusion strength and discrimination slopes before training (r = 0.11, p = 0.655). We found no significant main effects or interactions for any factor on the lapse rates, although the main effect of training approached significance (F(1,8) = 4.68, p = 0.062; all other p’s>0.171).

### Pooled samples

Power analysis suggested that based on the variance of illusion differences we estimated that we could detect a change in illusion strength of approximately 7° with 80% power at α = 0.05. Due to the noisier measurements and smaller sample size this was somewhat worse for experiment 1 (7.4°) than experiment 2 (6.1°). This corresponds closely to the mean of pre-training illusion magnitude in experiment 2 (6.9°) although it is in excess of the mean for experiment 1 (5.5°), presumably again due to the larger variability in PSEs in experiment 1. Nevertheless, this suggests that with our sample sizes it should have been possible to detect the learning effect predicted by an inherent relationship between illusion magnitude and discrimination ability.

However, more subtle reductions in illusion strength could be missed with the sample sizes we used. We therefore performed an additional analysis by pooling the data from the two experiments with a combined sample size of n = 17. While the two experiments used somewhat different procedures the magnitudes of the effects were largely consistent and the same experimental manipulations were tested. This analysis corroborated the main findings from the two experiments: for discrimination slopes there was a significant main effect of session and trained orientation and an interaction between these factors (Table 5). Using the pooled sample we estimate that with 80% power we could have detected changes in illusion magnitudes of 4.3°, which is substantially below the mean of pre-training illusion magnitudes (6.2°) or the theoretically relevant change (5–8°) if the linear relationship between discrimination ability and illusion strength were maintained after training. However, we found no significant main effects or interactions on illusion strengths/PSE except for a highly significant main effect of surround confirming that the tilt illusion was produced reliably across both experiments (Table 6).

**Table 5:** Three-way ANOVA on discrimination slopes pooled across experiments.

Factor/Interaction	Degrees of freedom	F	p
Surround	1, 16	3.53	0.079
Trained	1, 16	6.32	0.023*
Session	1, 16	11.91	0.003*
Surround×Trained	1, 16	0.10	0.753
Surround×Session	1, 16	0.05	0.822
Trained×Session	1, 16	5.65	0.030*
Surround×Trained×Session	1, 16	0.08	0.780

*p<0.05.

**Table 6:** Three-way ANOVA on PSE pooled across experiments.

Factor/Interaction	Degrees of freedom	F	p
Surround	1, 16	42.24	<0.001*
Trained	1, 16	0.33	0.573
Session	1, 16	0.44	0.516
Surround×Trained	1, 16	3.31	0.088
Surround×Session	1, 16	0.00	0.992
Trained×Session	1, 16	0.88	0.362
Surround×Trained×Session	1, 16	2.41	0.140

*p<0.05.

## Discussion

We examined a potential inherent trade-off between orientation discrimination sensitivity and the strength of the tilt illusion. In both experiments we showed that training for three days improved orientation discrimination ability at the trained orientations. If illusion strength changed directly with discrimination ability, this would suggest that they are both mediated by a common mechanism. Altering the function of neuronal connections that sharpen orientation tuning the output of neuronal populations giving rise to the illusion may also be affected by training. Another explanation could be that participants with poor discrimination ability tend to overestimate their subjective perceptual biases. However, in neither experiment was the magnitude of the tilt illusion reduced by training discrimination ability. Our results therefore suggest that discrimination sensitivity and illusion magnitude are not innately linked. Discriminability is presumably governed by factors other than illusion magnitude even though the two measures share a common relationship with V1 surface area and/or cortical magnification [4], [5].

Our previous work found that discrimination sensitivity and illusion magnitude are negatively correlated [4]. These factors were also linked to the large inter-individual variation in the surface area of V1 [19], [20], which had previously also been linked to other contextual visual illusions [21], [22]. Increased discrimination sensitivity and weaker illusion magnitude are observed in individuals with greater V1 surface areas, which may influence perception through the scaling of intra-cortical connections [4], [5]. One caveat to this finding is that in the present study we did not find a significant correlation between illusion magnitude and discrimination slopes although at least in experiment 1 we observed the same trend. However, this is unsurprising in light of the reduced statistical power for detecting such individual differences in our present sample. The trade-off between orientation discrimination sensitivity and tilt illusion magnitude may thus be a result of less global context-orientated in favor of local detail-orientated processing in individuals with larger V1. However, while tilt illusion magnitude may be stable and at least partly determined by V1 surface area (which is presumably also stable), orientation discrimination sensitivity is malleable by experience and presumably involves additional factors that can undergo lasting changes due to learning.

Thus our results also speak to a long-standing debate in the literature on whether perceptual learning directly affects neurons in the early stages of the visual processing hierarchy or whether it instead reflects reweighting in higher areas of *output* from the early stages of processing [23], [24], [25]. This latter interpretation contrasts with the traditional view of perceptual learning that it is mediated by early stages of the sensory processing pathway, where the receptive field properties and functional architecture have been well explored [26], [27], [28]. Perceptual learning is typically found to be specific to stimulus features such as position and orientation [29], [30], [31], [32] and such features are represented with fine resolution early in the visual processing pathway [33], [26], [34], [35]. However, using a feature and location double-training procedure eliminates this specificity and enables transfer of perceptual learning across both retinal locations and orientations [36], [24], [25]. This indicates that perceptual learning may not (only) occur through retuning of early visual neurons but rather involves a change in how the output of those neuronal populations is extracted by higher stages at the visual system, for example by altering the way locations in the visual field are attended [25].

The reverse hierarchy theory [23] of perceptual learning also predicts learning transfer indicative of higher level involvement in perceptual learning. Transfer suggests a modification of neurons with generalizing receptive fields, found at high cortical levels. Subsequently, higher visual areas are also thought to exert their effect on perceptual learning by enhancing the representations of stimuli in lower visual areas through a top-down process. This idea has found support in fMRI studies of the training of orientation discrimination [37], [38], [32], [39].

In contrast, the tilt illusion and the similar tilt aftereffect are more likely to arise from processing in early visual areas, such as V1 [40], [10], [41], [42]. The tilt illusion also occurs even when the surround is not consciously perceived [43] further implicating an early stage of visual processing. It may occur because V1 neurons have very localized receptive fields and are preferentially tuned to a specific orientation, but are inhibited by other, similar orientations. In the tilt illusion the neurons responding to the surrounding center presumably inhibit the neurons encoding the central target. At the population level this should manifest as a shift of the population tuning curve away from the orientation of the surround [44], [1], [45]. The dissociation we observed between experience-dependent improvements in discrimination and illusion magnitude suggests that perceptual learning may involve different, presumably higher level, processes to those responsible for the tilt illusion.

As part of our experiment 1 we re-tested orientation discrimination sensitivity and illusion magnitude approximately one month after the training ended. The improvement in discrimination ability remained stable over this period. One similar study [10] found a change in PSE (without a surround) reminiscent of the tilt aftereffect after six days of training orientation discrimination. Unlike the improvement of discrimination ability, this learning effect on PSE was not lasting but diminished even a week after training around a near-vertical orientation was completed. This suggests that continuous exposure to an orientation due to training may cause transient changes in the way orientation is encoded. There have been reports of changes in tuning curves measured for V1 neurons resulting from training orientation discrimination [28]; although such early changes remain controversial [46], changes in tuning properties of V1 neurons would predict changes in illusion magnitude. The training periods in such studies are far more extensive than the three day training we employed and changes in early visual neuronal responses may only arise with longer training periods. It is therefore possible that we could have observed a reduction in illusion strength with a much larger number of training sessions. Some investigations of perceptual learning train participants for many more sessions than we employed [30], [37]. Performance typically improves very steeply in the initial sessions and then levels out. The early phase of perceptual learning may thus involve more general improvements and higher level processes but later stages may become more specific and result in synaptic changes in early visual cortex. Moreover, it is also possible that by enhancing the statistical power of our design by training much larger groups of participants, we might have observed subtle changes in illusion magnitude after training. However, our results suggest that at least the amount of change expected by our previously reported relationship between illusion strength and discrimination ability does not occur even though discrimination improved significantly in our experiments.

Our results already demonstrate, however, that there is no *inherent* link between discrimination and illusion magnitude: improved discrimination did not result in a concomitant reduction in illusion strength. This argues against a trivial, artifactual relationship between these two factors. It also supports the interpretation that these two aspects of our perceptual experience are mediated by dissociable processes and may occur in different neuronal substrates. Even though both discrimination sensitivity and illusion magnitude may depend on neural substrates in early visual cortex, there are additional factors involved in learning discrimination.
